# Overweight in young males reduce fertility in rabbit model

**DOI:** 10.1371/journal.pone.0180679

**Published:** 2017-07-10

**Authors:** Francisco Marco-Jiménez, José Salvador Vicente

**Affiliations:** Institute of Science and Animal Technology, Laboratorio de Biotecnología de la Reproducción, Universidad Politécnica de Valencia, Valencia, Spain; University Hospital of Münster, GERMANY

## Abstract

Semen quality has certainly declined over the past few decades, possibly owing to modern lifestyle factors. In this sense, the role of overweight and obesity in the development of subfertility in males has generated a considerable amount of interest in recent years. However, there is no consensus on whether overweight or obesity impaired sperm quality. Thus, based on the ongoing debate about risk factors for subfertility associated with overweight and obesity in men, this study was designed to investigate the effect of overweight on sperm quality parameters and fertility success in randomized controlled trial in a rabbit model. Fourteen male rabbits were randomly assigned to a control group in which nutritional requirements were satisfied or a group fed to satiety from 12 to 32 weeks of age. At 24 weeks of age, semen samples were analysed weekly by conventional semen analysis for 8 weeks. In addition, during the trial female rabbits were artificially inseminated by each male to assess the fertility success and the number of offspring. Young males fed to satiety were associated with a significant increase in body weight (13.6% overweight) and perirenal fat thickness (5%). Male overweight presented a significant decrease in sperm concentration. There were no differences in the remaining sperm parameters. However, male overweight showed a clear and significant decrease in fertility success (control group, 64±8.9% *versus* fed to satiety group, 35±9.2%), but not in the number of offspring. Taken together, our findings provide new evidence on the loss of fertility induced by overweight in males.

## Introduction

Semen quality has certainly declined over the past few decades, possibly due to modern lifestyle factors [1]. In fact, around 49 million couples were infertile in 2010, and roughly 40–50% of infertility is caused by male factors [2–4]. The stand or fall of these situations depends on multi-factors i.e. genetic, environmental, behavioural or dietary [5]. According to the WHO, the worldwide prevalence of obesity in men has doubled between 1980 and 2008, increasing from 5% to 10% among men (BMI ≥30 kg/m2, [6]). In 2013, the proportion of men with overweight (body-mass index [BMI] of 25 kg/m2 or greater) has accelerated to an average of 36.9% [7]. As a result, there has been increasing research interest in studying the impact of BMI on sperm quality [5], partly because there are considerable numbers of overweight and obese infertile couples in their reproductive years [8]. However, it is still unclear to what extent overweight and obesity affects sperm quality [5,9]. In fact, the relatively limited data published are conflicting [1].

Many relevant research works published in succession, including meta-analyses, have described a negative association between BMI and different semen quality parameters (semen volume, sperm concentration, total sperm count, motility, normal sperm morphology, [5, 10–18]). However, these results have not been replicated in all reports on the topic [19–24]. Thus, we should be careful when interpreting the results, as they arise from cross-sectional studies or prospective population-based cohort studies on couples attempting to conceive, uncontrolled and non-randomized. In light of this limitation, animal models provide a viable alternative [25,26]. In fact, animal models have shown that the capacitation status and sperm binding ability of high fat diet mice were impaired [27–31]. In addition, it was established that male obesity reduced implantation and live birth rates [32,33]. Thus, based on the continuing debate about risk factors for subfertility associated with overweight and obesity, the present study tested the effects of overweight on sperm quality parameters and fertility in a randomized controlled trial in a rabbit model.

## Materials and methods

All chemicals, unless otherwise stated, were reagent-grade and purchased from Sigma-Aldrich Química S.A. (Alcobendas, Madrid, Spain). All the experimental procedures used in this study were performed in accordance with Directive 2010/63/EU EEC for animal experiments and reviewed and approved by the Ethical Committee for Experimentation with Animals of the Polytechnic University of Valencia, Spain (research code: 2015/VSC/PEA/00061).

### Animals

A total of fourteen males from a synthetic line of rabbits called line R were used in the experiment (S1 Fig). Line R comes from the fusion of two paternal lines, one founded in 1976 with Californian rabbits reared by Valencian farmers and another founded in 1981 with rabbits belonging to specialized paternal lines [34]. The selection method was individual selection on post-weaning daily gain, weaning taking place at 28 days and the end of the fattening at 63 days. The current generation of selection is the 36th. The rabbit has been used as an experimental animal in genetics and reproduction physiology since the turn of the century [35].

Animals were housed at the Polytechnic University of Valencia experimental farm in flat deck indoor cages (75×50×30 cm). The photoperiod is set to provide 16 h of light and 8 h of dark, and the room temperature is regulated to keep temperatures between 10°C and 28°C. Young males were fed to satiety until 12 weeks of age with a commercial pelleted diet (minimum of 15 g of crude protein per kg of dry matter (DM), 15 g of crude fibre per kg of DM, and 10.2 MJ of digestible energy per kg of DM). Until 9 weeks of age, young males were caged collectively, and subsequently housed individually. At 12 weeks of age, males were then randomly assigned to the control feed group in which nutritional requirements were satisfied, or the fed to satiety group. The control feeding regime consisted of a daily intake of 130 g to cover energy requirements for maintenance (340 kJ day-1 kg-1 LW0.75 [36]). The average daily feed intake of the fed to satiety group was determined by weighing the feeder at the beginning and end of each week, and all feed supplies given within a week were recorded. The feeding regime was maintained throughout the experiment.

### Semen quality assessment

Routine diagnostic semen analyses were carried out in this experiment. Semen samples were obtained by artificial vagina and collected into a sterile tube. Previously, males began the training period with an artificial vagina at 20 weeks of age (pubertal age [37]). Training was performed for 4 weeks and then males started the trial (24 weeks of age). For the training and production period, two ejaculates were collected per male and week on a single day using an artificial vagina, with a 30 min interval between collections. Only ejaculates that exhibited white colour were used in the experiment. Samples containing urine and cell debris were discarded while gel plugs were removed. Ejaculates from the same male each day were pooled as a single sample. Collections from each male were performed on the same day of the week for 8 weeks. A total of 112 semen samples were evaluated. The semen quality variables determined were: volume, sperm concentration, total sperm count, percent motility, sperm progressive motility, viability (integrity of the plasma membrane of the head), abnormal sperm and intact apical ridge (acrosomal status). The volume of the ejaculate was measured in a graduated conical tube. Aliquots from each ejaculate (20μl) were diluted 1:20 with Tris-citrate-glucose extender (250 mM tris-hydroxymethylaminomethane, 83mM citric acid, 50mM glucose, pH 6.8–7.0, 300 mOsmkg-1) to assess total motility and sperm progressive motility using an Integrated Semen Analysis System v. 1.0.17 (ISAS; Projectes i Serveis R+D S.L). The system was set to record images at 25 frames/s. Then, 10 μl of the sample was placed in a 10 μm deep Makler counting chamber. Motility was assessed at 37°C at 20x using a negative phase contrast microscope. For each sample, six microscopic fields were analysed and a minimum of 400 sperm evaluated. The same sample was assessed for the percentage of live and dead spermatozoa using the LIVE/DEAD sperm viability kit (Molecular Probes), which consists basically of two DNA-binding fluorescent stains: a membrane-permeant stain, SYBR-14, and a conventional dead-cell stain, propidium iodide [38]. Another aliquot was also diluted 1:20 with 0.5% of glutaraldehyde solution in phosphate buffered saline to calculate the concentration in a Thoma-Zeiss counting cell chamber and evaluate both the percentages of intact apical ridge [intact (AI) or reacted (AD)] and abnormal sperm (spermatozoa with morphological abnormalities of head, neck-midpiece and tail [AS]) by phase contrast at x400 magnification. The percentage of sperm with intact apical ridge was calculated as the ratio: [AI/(AI + AD)] x 100. The percentage of abnormal sperm (AS) was calculated as the ratio: [AS/(AI + AD + AS)] x 100.

### Pregnancy success and number of offspring

During the trial period, a total of 375 inseminations were carried out (189 in fed to satiety group and 186 in control group). The semen sample for each male was adjusted to a final sperm concentration of 20x10^6^ cells/ml with Tris-citrate-glucose extender. Finally, each female was inseminated with 0.5 mL (10x10^6^ cells per doe), which was performed within 2–4 h of semen collection. At insemination time, each female was injected intramuscularly with 1 μg of buserelin acetate (Hoechst Marion Roussel, S.A., Madrid, Spain) to induce ovulation. Only receptive does (red colour of vulvar lips) were inseminated, using a standard curved plastic pipette (Imporvet, S.A., Barcelona, Spain). The number of females that gave birth by number of inseminations (fertility rate) and number of offspring per litter were recorded.

### Body condition

Body weight and perirenal fat thickness (PFT) were measured across three testing sessions during the trial: at the beginning (24 weeks of age), in the middle (28 weeks of age) and at the last session (32 weeks of age). PFT was measured by ultrasound as described by Pascual et al. [39]. Briefly, images were obtained by a portable colour Doppler ultrasound device (Esaote, Spain) with 7.5 MHz linear probe (4–12 MHz range). PFT measures were indirectly obtained using the software of the ultrasound unit. Thus, at the moment of each PFT measurement all males were also weighed.

### Statistical analysis

The model used to examine the relation between daily food intake and semen parameters was a mixed model (SAS Institute Inc., Cary, NC, USA), according to a repeated measures design that takes into account the variation between animals and covariation within them. Covariance structures were objectively compared using the most severe criteria (Schwarz Bayesian criterion [40]). The model included the daily food intake (fed to satiety or restricted), as well as the time (week) and their inter-action as fixed effects. Random terms in the model included a permanent effect of each animal (p) and the error term (e), both assumed to have an average of zero and variance σ2p and σ2e, respectively. To evaluate the possible effect of DFI variability on semen characteristics, intra-animal coefficient of variation of DFI in four consecutive weeks was included as a covariate.

To compare fertility between groups, a general linear model was performed including the daily food intake regimen with two levels (control or fed to satiety) as a fixed factor. The error was designated as having a binomial distribution using the probit link function. Binomial data for fertility were assigned a value of one if AI was positive. Failure to inseminate resulted in a score of zero. Prolificacy was compared with a generalized linear model including the daily food intake regime with two levels (fed to satiety and control) as a fixed effect. Finally, body weight and perirenal fat thickness were compared using a T-test.

Differences of p<0.05 were considered significant. Data are shown as means ± standard error means (S.E.M.). All analyses, except mixed model (SAS Institute Inc., Cary, NC, USA) were performed with SPSS 21.0 software package (SPSS Inc., Chicago, Illinois, USA, 2002).

## Results

**Fig 1:**
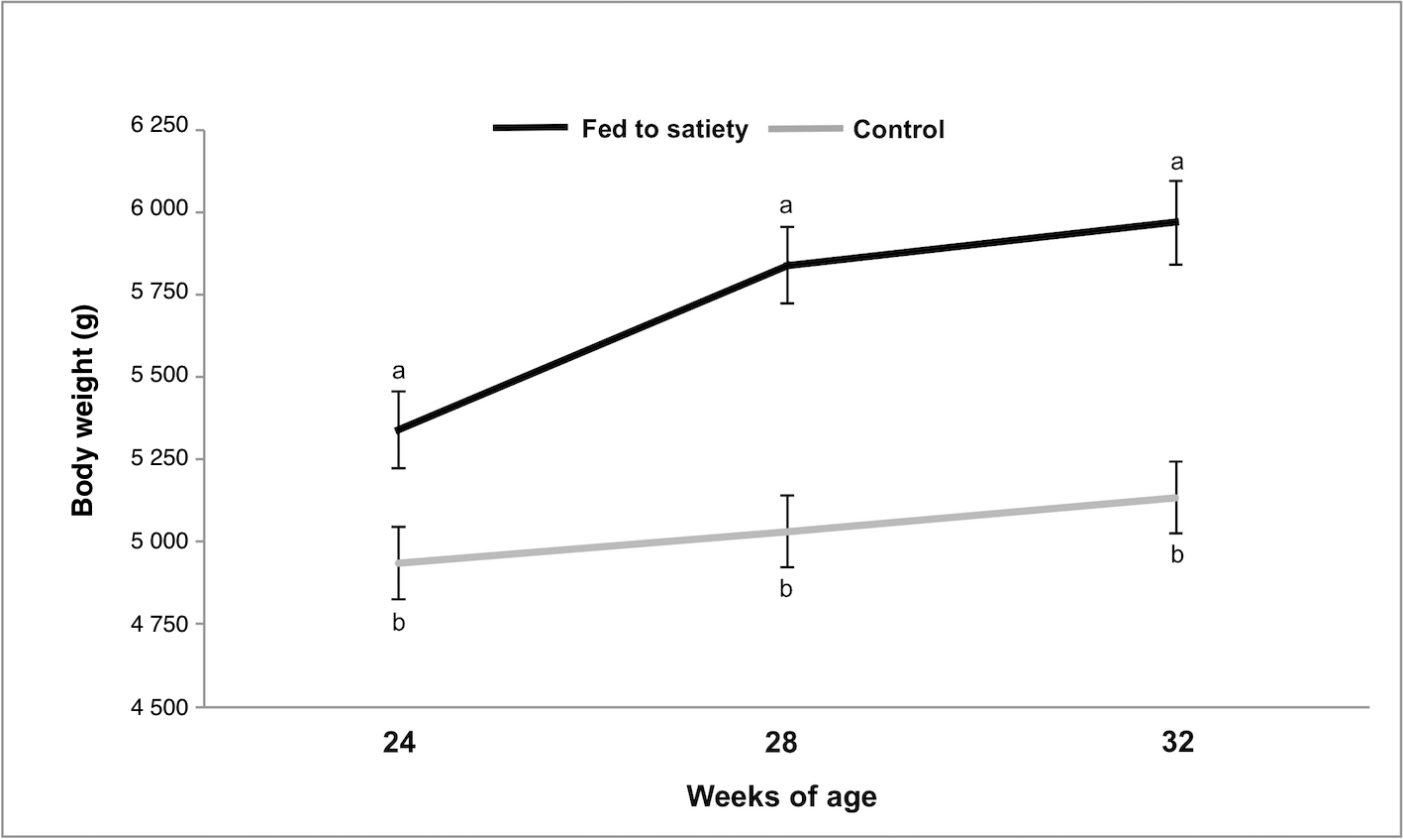
Effect of dietary treatment (fed to satiety and control to maintenance requirements) on the body weight evolution of rabbit males throughout the experiment. a,b Weeks not sharing letters are significantly different at P < 0.05.

**Fig 2:**
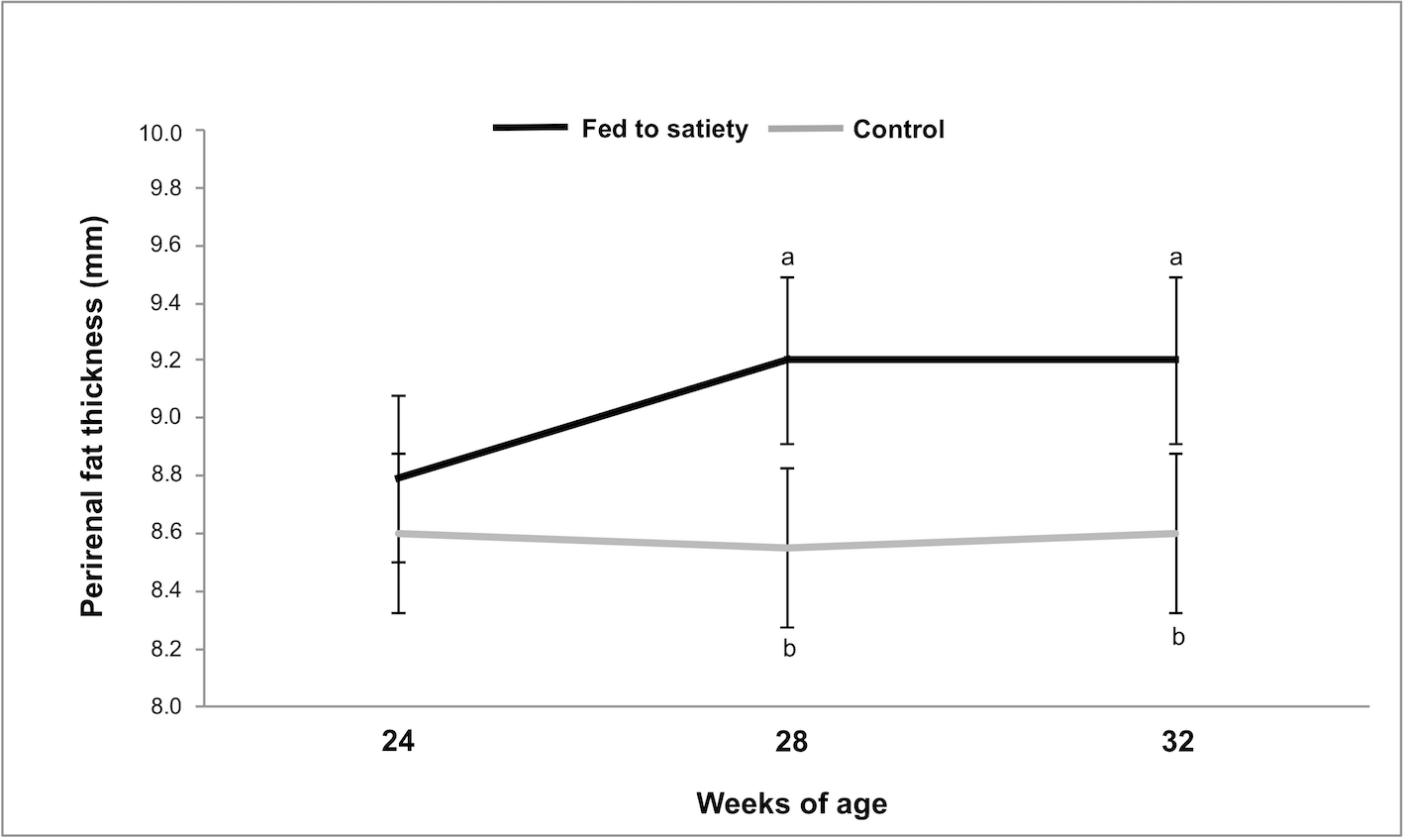
Effect of dietary treatment (fed to satiety and control to maintenance requirements) in the evolution of perirenal fat thickness of rabbit males throughout the experiment. a,b Weeks not sharing letters are significantly different at P < 0.05.

Males fed to satiety presented significantly increased daily feed intake of about +75% compared to restricted animals (227.5±6.87 g/day *versus* 130.0 g/day). In addition, young males fed to satiety were associated with a significant increase in body weight at the end of experimental period (control, 5 034.7±62.44 g *versus* fed to satiety, 5 717.8±69.66 g, p = 0.0001, Fig 1). As expected, the same tendency was reported for PFT (control, 8.60±0.159 mm *versus* fed to satiety, 9.03±0.178 mm, p = 0.0001, Fig 2).

All males were adapted to ejaculate into an artificial vagina. Based on its appearance, in the males fed to satiety 2 ejaculates were discarded due to the presence of urine, while in the control males, 2 ejaculates were discarded by urine contamination and 1 due to the presence of blood debris. Males fed to satiety had significantly lower sperm concentration (211.0±25.38 x10^6^/mL, p<0.05) when compared with normal weight (308.7±25.38 x10^6^/mL). There were no differences in the remaining sperm parameters among groups (Table 1). All the sperm parameters were within the normal range in rabbit.

**Table 1:** Effect of fed to satiety on sperm quality parameters in randomized controlled trial in rabbit model.

Semen quality variable	Control	Fed to satiety	P value
General characteristics			
Volume (ml)	0.7±0.10	0.8±0.10	0.091
Sperm concentration (10^6^/ml)	308.7±25.38^a^	211.0±25.38^b^	0.006
Total sperm count (x10^6^/ejaculate)	183.8±21.65	176.9±21.40	0.877
Sperm motility			
Percent motility (%)	66.3±3.48	56.4±3.43	0.059
Sperm progressive motility (%)	37.0±2.53	35.2±2.53	0.601
Integrity of the plasma membrane			
Viability (%)	85.9±1.86	81.6±1.86	0.116
Morphology			
Abnormal sperm (%)	21.5±2.01	26.4±2.03	0.119
Intact apical ridge (%)	87.6±1.52	84.0±1.50	0.103
Total males	7	7	

a,b superscript: Data in the same row with uncommon superscript are different (p<0.05). Data are presented as least squares means ± standard error of the least squares means.

Fed to satiety males showed a clear and significant decrease in fertility rate (64±8.9% *versus*. 35±9.2%, p<0.05. Table 2). However, no differences in total offspring and liveborn offspring were observed (7.1±0.32 and 5.9±0.36 *versus* 7.0±0.28 and 5.9±0.31, for control *versus* fed to satiety, respectively, Table 2).

**Table 2:** Effect of male fed to satiety on fertility and offspring in randomized controlled trial in rabbit model.

Treatment	N	Pregnancy success (%)	Total offspring	Live-born offspring
Control	186	64±8.9^a^	7.1±0.32	5.9±0.36
Fed to satiety	189	35±9.2^b^	7.0±0.28	5.9±0.31

N: total number of artificial inseminations.

a,b superscript: Data in the same column with uncommon letters are different (p<0.05). Data are presented as least squares means ± standard error of the least squares means.

## Discussion

In spite of the reports published during the last decade it is still unclear to what extent overweight/obesity affects sperm quality [5,9]. This control experiment demonstrates that being moderately overweight (13.6%) has a negative impact on the fertility rate, although conventional sperm quality parameters were not affected, except sperm concentration. The major concerns with case-control studies, as an observational design, are information bias, especially recall bias, and confounding [41–43]. In human studies, confounding factors associated with lifestyle and pathologies [30] could partly explain the lack of consensus on the relationship between overweight/obesity and male fertility [44].

Our results showed a significant relationship between overweight and sperm concentration. Specifically, overweight males exhibited lower sperm concentration than restricted animals, in accordance with previous studies [5, 13, 45–47]. However, some studies failed to report any association between BMI and sperm concentration [44, 48,49] or found, in a meta-analysis, an inverse relationship only in subfertile men [20]. We did not observe a significant relationship between overweight and the rest of the sperm assessment. The result was consistent with previous relevant meta-analysis, which found no relation between BMI and sperm parameters [12,20]. In contrast, our calculation drew a different result to the previous relevant reports [11,13,50], including an updated meta-analysis in 2017 [5]. It therefore remains to be determined if overweight (BMI>25) and obesity (BMI>30) have a negative impact on sperm quality and therefore on fertility [5, 51]. Overweight has been associated with endocrine disturbance regarding the decreased level of sex hormone binding globulin and testosterone levels and a concomitant increased plasma concentration of oestrogen [20, 51, 52], changes in spermatogenesis and Sertoli cell function, and an increase in scrotal temperature [12, 51]. Taken together, these findings suggest that the effects of male overweight/obesity on sperm quality and fertility are likely multifactorial [1, 51]. As an example, the proposed pathophysiological mechanisms underlying these sophisticated relationships have been discussed in areas varying from endocrinology to psychology [5]. We suggest that in part the lack of consensus, besides multifactorial response, would be explained by the clinical limitations of BMI. One particular issue with BMI as an obesity index is that it fails to distinguish between fat mass at different body sites [53, 54]. Likewise, the accuracy of the BMI in determining body fat mass has frequently been questioned, as it is clearly limited for this purpose [54].

Despite all the aforementioned data, and although the results of the present study do not support the idea of a deleterious effect of a moderated overweight on conventional semen parameters (except sperm concentration), the most interesting finding in this work was that overfeeding was related to subsequent overweight, resulting in a fertility rate decreased by half. A similar effect has been described in infertile couples undergoing assisted reproductive technology, with lower odds of live birth after in vitro fertilization treatment compared with intracytoplasmic sperm injection (ICSI) among overweight and obese men, supporting the hypothesis that ICSI may overcome a possible obesity-related impairment of the sperm-oocyte interaction [55]. Our data can be considered conclusive based on the high number of inseminations. Furthermore, daily feed intake was the only source of variation in our model. In this sense, when males ate to satiety for a short-term period, it significantly increased body weight and perirenal fat deposits. It is known that correlation between semen quality and fecundation is complicated, and no accepted threshold values exist for any semen variables on an individual level [1]. In fact, conventional semen parameters fail to provide accurate measurements and only give partial information on sperm functions [12]. Similarly, sperm parameter cut-off values have also been cited as being of insufficient clinical relevance due to variations in the results of semen analysis, related with physiological variations and the limitations of the techniques applied [56]. In animal models, feeding with a high-fat diet was associated with deleterious changes in volume, sperm count, sperm morphology, sperm motility and fertility [28–31]. Although direct comparison among these studies must be applied carefully owing to their inherent experimental differences (specie, genotypes, others), the differences in semen parameters could be caused by the light degree of overweight/obesity experienced in our experiment when compared to the administration of high-fat diet. The detrimental effect of hypercholesterolemia on Leydig and Sertoli cell secretory function, spermatogenesis, epididymal sperm maturation process, and the overall sperm fertilizing capacity has been described in the few past decades [29, 57–59]. Most importantly, and consistent with these previous studies, is our result that the fertilizing capacity of male overweight was negatively affected. Recently, several studies have indicated that overweight/obesity status alters protamination and microRNA abundance, as well as changing the epigenetic status in sperm cells, which may explain in part the loss of fertilizing capacity and the outcome of the progeny [51, 60–62].

## Conclusions

In conclusion, the present study reports that sperm quality parameters analysed by conventional semen analysis, except sperm concentration, are not associated with male overweight in our rabbit model. However, our data provide evidence that overweight is related to a clear reduction in fertility capacity. Thus, future studies contrasting the “genomic” and “proteomic” profiles of sperm cells are warranted to identify the specific changes that could impair sperm fertility capacity in overweight/obese males.

## Supporting information

S1 Fig Rabbit of a synthetic line called line R. (TIFF)
