# Correction: Overweight in young males reduce fertility in rabbit model

**DOI:** 10.1371/journal.pone.0209378

**Published:** 2018-12-12

**Authors:** Francisco Marco-Jiménez, José Salvador Vicente

The name of the institution is spelled incorrectly in the affiliation for authors Francisco Marco-Jiménez and José Salvador Vicente. The affiliation should be: Institute of Science and Animal Technology, Laboratorio de Biotecnología de la Reproducción, Universitat Politècnica de València, 46022 Valencia, Spain.
