# Supplementary figures and images for: Overweight in young males reduce fertility in rabbit model
Source: PLoS One. 2017 Jul 10;12(7):e0180679. doi: 10.1371/journal.pone.0180679 (PMC5503274; doi:10.1371/journal.pone.0180679)

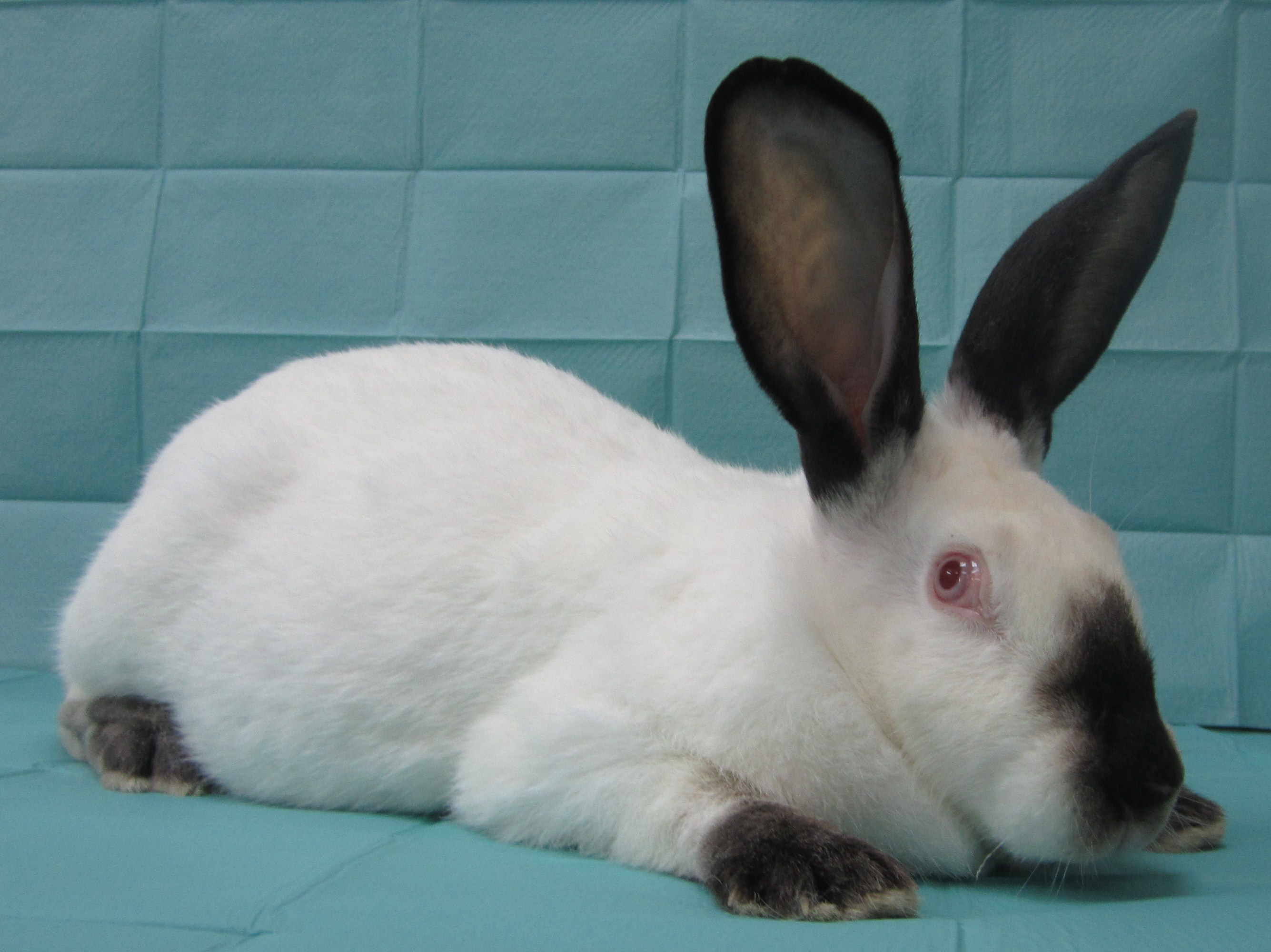

Supplement: S1 Fig — (TIFF) [file pone.0180679.s001.tiff]
